# Rethinking host range in *Pneumocystis*

**DOI:** 10.1371/journal.ppat.1008824

**Published:** 2020-09-10

**Authors:** Spenser J. Babb-Biernacki, Jacob A. Esselstyn, Vinson P. Doyle

**Affiliations:** 1 Museum of Natural Science and Department of Biological Sciences, Louisiana State University, Baton Rouge, Louisiana, United States of America; 2 Department of Plant Pathology and Crop Physiology, Louisiana State University AgCenter, Baton Rouge, Louisiana, United States of America; University of Maryland, Baltimore, UNITED STATES

## Introduction

Fungi in the genus *Pneumocystis* are obligate biotrophs and opportunistic pathogens of mammal lungs. They are notable for their high degree of host specificity; it is often argued that *Pneumocystis* species are restricted to a single host species (i.e., monoxenous) [1, 2]. This monoxenous hypothesis, combined with their ubiquitous presence across mammal diversity, suggests that there may be one *Pneumocystis* species for each of the 6399 extant mammal species. However, only a small percentage of mammal species have been tested for *Pneumocystis*, and only five *Pneumocystis* species have been formally described. Despite our massive knowledge gaps, the notion of monoxenism in *Pneumocystis* is so pervasive that evidence to the contrary is often described as rare exceptions to a rule [1, 2]. Here, we review the literature on host distribution of *Pneumocystis* and argue that monoxenism is not supported by the available, albeit limited, data. We emphasize the importance of species discovery and studies of *Pneumocystis* host range as a prerequisite to the pursuit of other biological questions in these and other medically important fungi.

### Crossinfection experiments

**Fig 1:**
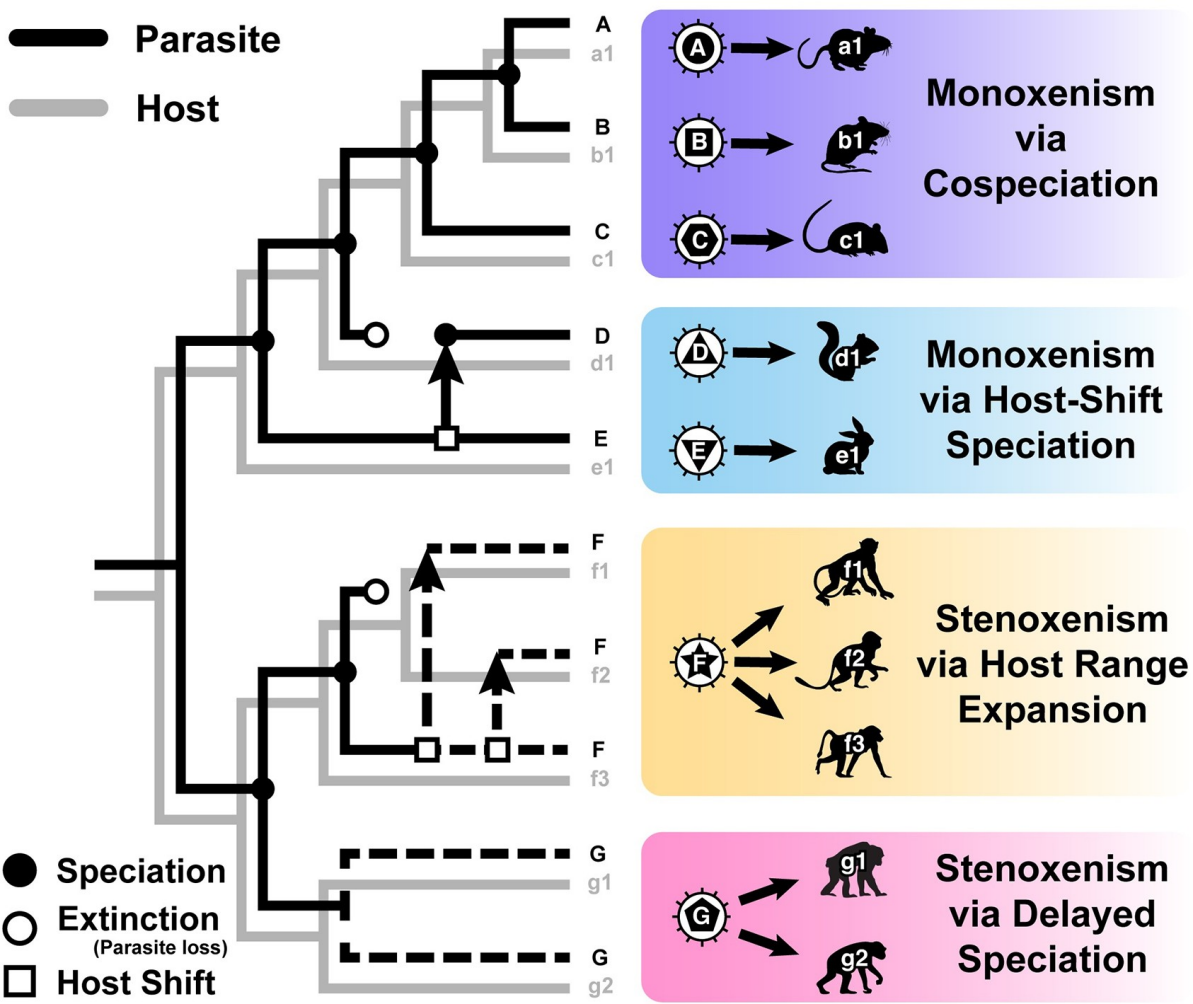
Various parasite host range hypotheses. Hypothetical host and parasite phylogenies illustrating scenarios that can lead to two forms of host specificity: monoxenism and stenoxenism. Dotted branches indicate a single parasite species residing in multiple host species. These patterns may all occur in a single parasite clade, such as Pneumocystis, and distinguishing between them requires thorough host sampling and robust phylogenetic analysis.

Our primary interest here is *Pneumocystis* host range: Are *Pneumocystis* species monoxenous (which is the dominant perspective) or are they commonly stenoxenous (inhabiting a narrow range of multiple host species; Fig 1)? To understand the host niche of any symbiont, we must identify species boundaries. Genealogical concordance phylogenetic species recognition (GCPSR), which interprets transitions between concordance and discordance of multiple gene trees as species boundaries [3], is a widely applied, well-supported method to recognize fungal species. However, the difficulty of sequencing multiple unlinked, nuclear loci in samples with low fungal loads has largely precluded the application of GCPSR to *Pneumocystis*. Our understanding of *Pneumocystis* species boundaries is thus informed by other recognition methods, historically beginning with crossinfection experiments.

From the discovery of *Pneumocystis carinii* in 1912 until 1976, *Pneumocystis* was thought to be a single species capable of living in a variety of mammals [4]. Morphological and immunological work [5] as well as crossinfection experiments challenged this. In 1993, researchers failed to infect laboratory mice with *Pneumocystis* from ferrets [6], suggesting that ferret-inhabiting *Pneumocystis* is distinct from the species known from mice. This failure of heterospecific transmission was soon observed in reciprocal transmission experiments among laboratory mice (*Mus musculus*), rats (*Rattus norvegicus*), and rabbits (*Oryctolagus cuniculus*) [7] and from humans to owl monkeys (*Aotus nancymaae*) [8], all species known at the time to host *Pneumocystis*. In a case used to argue for strong host specificity outside the laboratory, *Pneumocystis* was not transmitted between cohoused Egyptian fruit bats (*Rousettus aegyptiacus)* and Rodrigues flying foxes (*Pteropus rodricensis)* in a zoo [9]. These results led researchers to conclude that *Pneumocystis* is a diverse genus of host-specific species, but the host pairs tested for crossinfection are distant relatives that have been evolving independently for many millions of years.

These experiments demonstrated that *Pneumocystis* species exhibit host specificity, but because transfer experiments used distantly related hosts, the true degree of this specificity remains untested. The most closely related hosts involved in these transmission experiments, *Mus* and *Rattus*, are in the same subfamily (Murinae), but they are separated by approximately 11 million years of evolution [10]. More closely related hosts may share *Pneumocystis* species because generic or tribal host affinity is common in many pathogenic fungi [11]. Transmission attempts between closely related host species could reveal the true specificity of *Pneumocystis*, but sustaining laboratory populations of wild animals is often infeasible, and host specificity may also vary widely within *Pneumocystis*. As such, examining many natural mammal populations is necessary to assess host specificity.

### Discovery of multihost associated *Pneumocystis*

Compelling genetic evidence has emerged that *Pneumocystis* species occupy host niches broader than are commonly appreciated. In 2004, Guillot and colleagues [12] studied *Pneumocystis* in rhesus (*Macaca mulatta*) and long-tailed (*M*. *fascicularis*) macaques. Both species were infected with *Pneumocystis* organisms identical in sequence at the mitochondrial large subunit (mtLSU) locus, implying that they harbor the same *Pneumocystis* species [12]. Very similar *Pneumocystis* sequences (uncorrected *p*-distance < 1%; Table 1) that may belong to the same species have since been recovered from the southern pig-tailed macaque (*Macaca nemestrina*) [13]. Several additional examples of crosshost *Pneumocystis* come from rodents. These include two species of Eurasian field mice, *Apodemus sylvaticus* and *A*. *flavicollis*, which carried phylogenetically indistinguishable *Pneumocystis* [14]. Most striking is a study exploring *Pneumocystis* in wild populations of Southeast Asian rodents, which included excellent sampling from many congeneric species in related genera [15]. *Pneumocystis carinii* and *P*. *wakefieldiae* were detected in several species of *Rattus*, which challenged the belief that these species could only colonize *Rattus norvegicus*). More surprisingly, the red spiny rat (*Maxomys surifer)* and Herbert’s giant rat (*Leopoldamys herberti)*, which are separated by approximately 7 million years of evolution [10], were found to harbor identical *Pneumocystis* genetic sequences [15]. Nevertheless, the notion of monoxenism has persisted, and cases such as these are described as rare potential exceptions [1, 2]. However, these findings suggest that host range is constrained by host divergence time rather than the speciation process per se, which leads us to scrutinize the evidence used to conclude that *Pneumocystis* species are primarily monoxenous.

**Table 1:** Genetic distances (expressed as percentages) of mtLSU and mtSSU between *Pneumocystis* from closely related hosts.

mtLSU *p-*distances	1	2	3	4	5	mtSSU *p*-distances	1	2	3
1. *Callithrix jacchus* AF362454						1. *Callithrix jacchus* AF395577			
2. *Callithrix geoffroyi* AF362456	1.27					2. *Callithrix geoffroyi* AF395578	0		
1. *Allenopithecus nigroviridis* AF362464						1. *Cercopithecus hamlyni* AF395575			
2. *Cercopithecus hamlyni* AF362457	1.47					2. *Cercopithecus nictitans* AF395576	14.34		
3. *Cercopithecus nictitans* AF362460	2.56	1.84							
4. *Cercopithecus lhoesti* AY265382	1.54	1.16	1.54			1. *Callimico goeldii* AF395582			
						2. *Saguinus fuscicollis* AF395580	1.11		
1. *Callimico goeldii* AF362461						3. *Saguinus midas* AF395579	9.78	10.34	
2. *Saguinus fuscicollis* AF362462	1.12					4. *Saguinus oedipus* AF395581	10.85	11.44	3.22
3. *Sagunius midas* AF362455	5.26	4.14							
4. *Saguinus imperator* AF362465	7.73	6.44	1.71			1. *Macaca fascicularis* AF395574			
*5*. *Saguinus oedipus* AF362453	7.63	6.36	3.80	2.14		2. *Macaca mulatta* AF395573	1.38		
1. *Macaca nemestrina* AF362466						1. *Plecotus auritus* JQ061307			
2. *Macaca nemestrina* AY265383	0.82					2. *Plecotus austriacus* JQ061308	0.49		
3. *Macaca fascicularis* AF362466	0.39	1.24							
4. *Macaca fascicularis* AY265385	1.24	0.74	1.65						
5. *Macaca mulatta* AF402690	1.94	2.21	2.34	2.94		*p*-distance < 2% (common *Pneumocystis* intraspecific variation)			
6. *Macaca mulatta* AF402691	1.98	1.50	2.37	2.25	0.8	2% < *p*-distance < 4% (seen in some hypothesized species)			

Uncorrected *p*-distances (recalculated for this publication; pairwise gaps deleted) in mtLSU and mtSSU genetic sequences between *Pneumocystis* found in congeneric and other closely related hosts. mtLSU, mitochondrial large subunit; mtSSU, mitochondrial small subunit

### Expected divergence at *Pneumocystis* barcodes

As previously mentioned, the difficulty of sequencing nuclear loci from *Pneumocystis* in wild animals has prevented robust study of species boundaries. Two easily sequenced mitochondrial loci, the mitochondrial large subunit (mtLSU) and small subunit (mtSSU), have thus become de facto *Pneumocystis* barcodes [14,16]. Although single-locus studies are subject to the idiosyncrasies (e.g., introgression and strong selection) of small sample sizes [17], these loci are the only data available from many undescribed *Pneumocystis* species [e.g. 9, 15, 18] and are often the basis of claims about monoxenism. Therefore, we must critically examine how mtLSU and mtSSU variation has been used to understand species boundaries in *Pneumocystis*.

One problem to consider when using mtLSU and mtSSU as *Pneumocystis* barcodes is that, although the “barcode gap” is considered indicative of plausible species boundaries, we do not know how much inter- and intraspecific distance to expect in *Pneumocystis*. Here, we summarize the few available examples of intraspecific genetic sampling of *Pneumocystis* from multiple localities across a host range. In two well-studied *Pneumocystis* species, the human-associated *P*. *jirovecii* and mouse (*Mus musculus)*-associated *P*. *murina*, variation has been observed at only two or three bases out of the approximately 250 bp long mtLSU fragment (0.8% to 1.2%) [19, 20]. *Pneumocystis* sequences were recovered from populations of Mexican free-tailed bats (*Tadarida brasiliensis)* from Mexico and Argentina, with maximum divergence of 0.78% at mtLSU and 1.83% at mtSSU; in the common pipistrelle bat (*Pipistrellus pipistrellus)*, no divergence was observed among individuals at mtLSU, but 0.49% divergence was seen at mtSSU [9]. In Finnish and English populations of the common shrew (*Sorex araneus*), *Pneumocystis* mtLSU was 0.89% divergent [21]. These results suggest genetic variation at these loci within *Pneumocystis* species is generally lower than 2% and that samples exhibiting higher divergence may represent distinct species.

However, findings from wild populations of the genus *Rattus* challenge this. Comparatively high levels of divergence were identified in mtLSU sequences from wild *P*. *wakefieldiae* (up to 3.82%) and *P*. *carinii* (up to 1.95%) as well as mtSSU in *P*. *wakefieldiae* (up to 2.78%; uncorrected *p*-distances were not included in original study and calculated for this publication) across several Southeast Asian countries, much higher diversity than had ever been observed in these species in the lab [15]. Comparable levels of genetic variation were recovered from *Pneumocystis* found in the wood mouse (*Apodemus sylvaticus*) across its European range: up to 3.8% in a concatenated mtLSU and mtSSU alignment [18]. The cases of bats, shrews, and wood mice demonstrate that geographically isolated *Pneumocystis* populations can exhibit marked divergence that may reflect the phylogeography of their hosts.

*Pneumocystis* from other host species present striking heterogeneity at mtLSU and mtSSU. A diverse population of *Pneumocystis* was discovered in laboratory macaques (*Macaca mulatta* and *M*. *fascicularis*), possibly representing two species. Mean divergence within these two *Pneumocystis* clades was reported as 2.5% and 2.3% [12], respectively, but individual pairwise intraclade distances reached 5.0% by our *p*-distance calculations. Pig (*Sus scrofa domesticus*)-associated *Pneumocystis* is another heterogenous population in which mtLSU and mtSSU pairwise divergence ranges from 0.5% to over 15%, with no readily discernible phylogenetic clusters which could represent species boundaries (i.e., no barcode gap) [22]. Macaque and pig-associated *Pneumocystis* thus require study of multiple nuclear genes to locate plausible species boundaries. These examples demonstrate that without additional data, a clear, consistent barcode gap cannot yet be known for *Pneumocystis*, complicating our ability to interpret mtLSU and mtSSU variation as evidence of species boundaries.

### Reexamination of cospeciation

Because strong host specificity drives cospeciation in host-symbiont assemblages [23], the inference of significant cospeciation between *Pneumocystis* and their mammal hosts has been used to support the monoxenous hypothesis. However, successful cophylogenetic analysis requires accurate delimitation of symbiont species, independent of host identity. Oversplitting symbiont species using host identity leads to overestimation of cospeciation [24] and host specificity. In the case of *Pneumocystis*, cophylogenies have largely been inferred that include undescribed putative *Pneumocystis* species, with species assignments based on mitochondrial divergence and host identity. Here, we revisit the evidence used for *Pneumocystis* species assignment in two influential cophylogeny studies.

Important *Pneumocystis* research in primates and bats demonstrated significant host-symbiont phylogenetic concordance, but conclusions about host specificity based on these findings require a better understanding of species limits in *Pneumocystis*. In a study of 18 primate species, every host taxon harbored *Pneumocystis* with unique mtLSU sequences, which was taken as evidence that primate-associated *Pneumocystis* are monoxenous [13]. However, unique genetic sequences do not necessarily represent unique species, and many *Pneumocystis* organisms from closely related primates exhibit extreme similarity at mtLSU and mtSSU that may reflect intraspecific variation. A noteworthy example are the marmosets (genus *Callithrix*). *Pneumocystis* from the common marmoset (*Callithrix jacchus*) and white-headed marmoset (*C*. *geoffroyi*), which differ by only 1.27% at mtLSU by our *p*-distance calculations and are identical at mtSSU (Table 1). Low levels of divergence between *Pneumocystis* from other primate hosts (Table 1) questions the interpretation of separate species, because isolation by distance can occur in *Pneumocystis* from allopatric host populations, as previously established. The same phenomenon is observed in bats [9], often cited as the best example of highly host-specific *Pneumocystis* [e.g. 2, 15, 18]. In the only instance of successful intrageneric *Pneumocystis* sampling in bats, mtSSU was sequenced from two species of long-eared bat, *Plecotus auritus* and *P*. *austriacus*, and found to be only 0.49% divergent [9]. This is less than the intraspecific variation observed at mtSSU collected from *Pneumocystis* in the Mexican free-tailed bat (1.83%). Without further investigation, it is inappropriate to assume that slightly divergent sequences from two *Plecotus* species belong to unique *Pneumocystis* species, while more divergent sequences from the Mexican free-tailed bat represent the same *Pneumocystis* species.

In both primates and bats, the combination of limited sampling and similarity of *Pneumocystis* sequences from closely related hosts demands more rigorous assessment before drawing conclusions about cospeciation and host specificity. It seems no coincidence that bat-associated *Pneumocystis* is regarded as the best example of highly host-specific organisms, while also being the group with the least data from congeneric hosts. This recapitulates our primary criticism of crossinfection experiments: Because closely related hosts are not well sampled, the inferred monoxenism of *Pneumocystis* may be a sampling artifact.

Additionally, most *Pneumocystis* cophylogeny analyses have yet to test synchrony of host and *Pneumocystis* speciation, a requisite characteristic of true cospeciation. The emergence of *Pneumocystis* genomic data is changing this, as analysis of several genomes suggests that *Pneumocystis* species fail to diverge simultaneously with their hosts [25]. This is consistent with the more holistic view that *Pneumocystis* are generally stenoxenous, persisting in multiple descendants of their ancestral hosts.

Cospeciation analyses will be useful only in clades with robust species boundaries for both hosts and parasites. To achieve this, we recommend a perspective shift toward a new null hypothesis: Similar *Pneumocystis* populations belong to the same species, even if they are found in heterospecific hosts. Tests of this null hypothesis should employ data streams independent of host identity.

## Conclusions

The question of monoxenous versus stenoxenous host range in *Pneumocystis* is not merely semantic; the community’s understanding of host niche in *Pneumocystis* evolution has profound impacts on our understanding of their ecology and transmission potential [24, 26]. Overestimating cospeciation and host specificity can lead researchers to discount the role of host switching in a parasite’s evolutionary history, as has happened with some lineages of *Pneumocystis* that switched between rodent subfamilies [27] and between pigs and carnivorans [28]. Since most emerging diseases in humans result from zoonotic host switches [29], understanding past rates of host switches is a critical priority.

Certainly, these data are incomplete and reveal no hard truths about *Pneumocystis* host specificity, especially given the challenges of basing hypotheses on limited mitochondrial data from a limited number of host species. However, no experimental or genetic evidence has conclusively demonstrated single-host specificity in *Pneumocystis*, although this claim continues to be repeated [1, 2]. *Pneumocystis* is poorly sampled, with thousands of mammal host species untested; that we have already encountered many probable exceptions to monoxenism suggests that multihost range is not rare. The evolution of *Pneumocystis* host niche is certainly complex, as a recent study in African rodents suggested a mix of monoxenous and stenoxenous *Pneumocystis* species and several host switching events [27].

Echoing others [2], we urge researchers to focus on collecting more sequence data from *Pneumocystis* from more host taxa. Sequences from nuclear loci will be particularly important and will allow us to move past barcoding-based species hypotheses, enable formal identification of *Pneumocystis* species, and give us a deeper understanding of how fungal species boundaries correspond to those of their hosts. Of course, we acknowledge that this is a challenge in wild animal specimens with low *Pneumocystis* loads. Still, it is necessary if we are to understand the evolutionary history of these important fungi.
